# The long non-coding RNA *PCA3*: an update of its functions and clinical applications as a biomarker in prostate cancer

**DOI:** 10.18632/oncotarget.27284

**Published:** 2019-11-12

**Authors:** Ana Emília Goulart Lemos, Aline da Rocha Matos, Luciana Bueno Ferreira, Etel Rodrigues Pereira Gimba

**Affiliations:** ^1^Departamento de Epidemiologia e Métodos Quantitativos em Saúde, Escola Nacional de Saúde Pública/Fundação Oswaldo Cruz (FIOCRUZ), Rio de Janeiro, Brazil; ^2^Programa de Pós-Graduação em Ciências Biomédicas – Fisiologia e Farmacologia, Universidade Federal Fluminense, Rio de Janeiro, Brazil; ^3^Laboratório de Vírus Respiratórios e do Sarampo, Instituto Oswaldo Cruz, Fiocruz, Rio de Janeiro, Brazil; ^4^Coordenação de Pesquisa, Instituto Nacional do Câncer, Rio de Janeiro, Brazil; ^5^Departamento de Ciências da Natureza (RCN), Instituto de Humanidades e Saúde, Universidade Federal Fluminense, Rio de Janeiro, Brazil

**Keywords:** *PCA3*, prostate cancer, biomarker, long non-coding RNA (lncRNA), clinical applications

## Abstract

Prostate cancer antigen 3 (*PCA3*) is an overexpressed prostate long non-coding RNA (lncRNA), transcribed from an intronic region at the long arm of human chromosome 9q21–22. It has been described that *PCA3* modulates prostate cancer (PCa) cell survival through modulating androgen receptor (AR) signaling, besides controlling the expression of several androgen responsive and cancer-related genes, including epithelial–mesenchymal transition (EMT) markers and those regulating gene expression and cell signaling. Also, *PCA3* urine levels have been successfully used as a PCa diagnostic biomarker. In this review, we have highlighted recent findings regarding *PCA3*, addressing its gene structure, putative applications as a biomarker, a proposed origin of this lncRNA, roles in PCa biology and expression patterns. We also updated data regarding *PCA3* interactions with cancer-related miRNAs and expression in other tissues and diseases beyond the prostate. Altogether, literature data indicate aberrant expression and dysregulated activity of *PCA3*, suggesting *PCA3* as a promising relevant target that should be even further evaluated on its applicability for PCa detection and management.

## INTRODUCTION

Prostate cancer antigen 3 (*PCA3*) is long non-coding RNA (lncRNA), first described by using differential display experimental approach and named as *DD3* [1]. LncRNAs play key roles in a wide repertoire of biological processes by controlling gene expression and their dysregulation has been related to tumor progression [2].

*PCA3* is the most specific prostate cancer (PCa) molecule identified to date [1, 3]. It was identified in 1999, when it was reported as overexpressed in PCa, as compared to non-malignant prostate samples, in addition to presenting a prostate-specific expression pattern [1]. Since its first description, *PCA3* roles have been investigated in PCa and its applicability as a PCa specific biomarker has also been explored.

This review presents updated information regarding the characterization of this lncRNA since its first description. Consolidation of these data can open new avenues to investigate its role in PCa biology and future effective applications as a biomarker and a therapeutic target.

## *PCA3* GENE STRUCTURE

**Figure 1:**
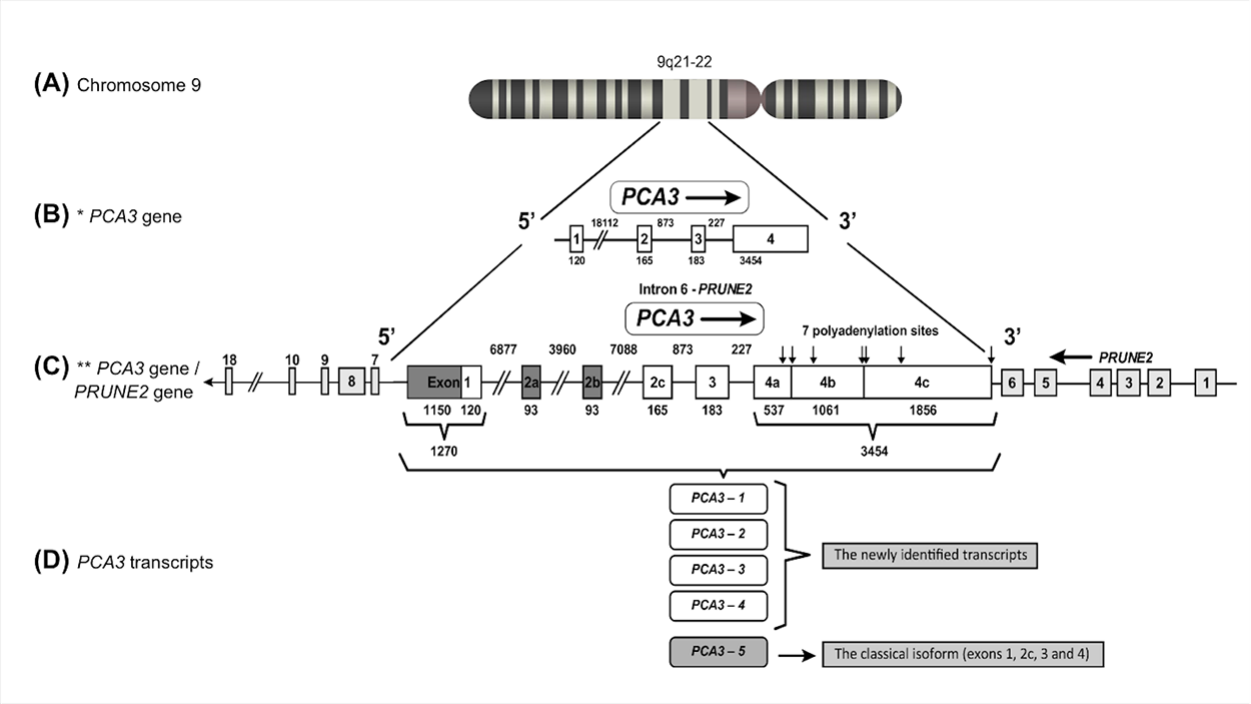
*PCA3* gene localization, structure and transcripts. **(A)** The *PCA3* gene is located on human chromosome 9q21–22. **(B)** The former *PCA3* gene structure [1], comprising 4 exons. **(C)** Updated *PCA3* gene structure [6], presenting a more complex transcriptional unit, including additional exons. In this description, exon 1 was found to be 1150 bp longer, comprising 1270 bp. Three alternative spliced exons were described in exon 2 (2a, 2b and 2c) and four additional polyadenylation sites were observed in exon 4, bringing the total number of polyadenylation sites to seven (indicated by vertical arrows). Dark boxes represent the most recently identified *PCA3* gene regions, which have 6 exons with alternative splicing of exon 2a (93 bp), 2b (93 bp) and 2c (original exon 2 was 165 bp). *PCA3* gene is embedded within the intron 6 of *PRUNE2* (also called *BMCC1* isoform 1). Light gray boxes represent *PRUNE2* exons and white boxes represent *PCA3* exons. These two genes are in the opposite orientation. **(D)** *PCA3* transcripts: *PCA3* isoforms 1-4 (the more recently identified transcripts by Clarke et al in 2009) and *PCA3*-5 (the classical isoform).

The first description of *PCA3* gene unit reported its location on human chromosome 9q21–22 (Figure 1A) and its 25 kb length containing four exons (Figure 1B) [1]. According to this report, the primary *PCA3* transcript can be submitted to alternative splicing, alternative polyadenylation and produces different sized transcripts. The classical isoform (termed as *PCA3*-5) contains exons 1, 3, 4a and 4b (Figure 1D). Moreover, the high frequency of stop codons detected in all *PCA3* reading frames further evidenced it as a non-coding RNA and no protein or peptide was found to be coded by *PCA3* transcripts. The nuclear localization of *PCA3* polyadenylated transcripts was demonstrated [3]. However, later reports also showed *PCA3* detection into the cytoplasm [4, 5].

More recently, a further detailed description of the *PCA3* gene structure was performed, presenting a more complex transcriptional unit, including novel additional exons (Figure 1C) [6]. Exon 1 was found to be 1150 bp longer, with 5 possible transcription start sites. Three variants were also described in exon 2 (2a, 2b and 2c) and four additional polyadenylation sites in exon 4 were observed, bringing the total number of polyadenylation sites to 7 [6] (Figure 1C). Besides, four supplementary ORFs were described at upstream regions of the original *PCA3* transcript. Furthermore, detailed investigations confirmed that no predicted peptide was coded by any *PCA3* transcript [7].

Additional *PCA3* isoforms have also been reported, named as *PCA3* isoforms 1-4 (Figure 1D), with transcription start sites respectively located at 1150 bp, 699 bp, 640 bp and 136 bp upstream from the original *PCA3* start site [6]. *PCA3-4* corresponds to only 1% of total *PCA3* transcripts, whereas the *PCA3-5* is the major transcript found in PCa tissue samples [1, 7] (Figure 1D).

Further investigations on the organization and evolution of the *PCA3* gene locus demonstrated that *PCA3* is an intronic antisense transcript, mapped in the opposite orientation of the Prune homolog 2 coding (*PRUNE2*) gene, also called *BMCC1* isoform 1 (*BMCC1-1*), within its intron 6 [6] (Figure 1C).

### *PCA3* origin

An interesting hypothesis regarding *PCA3* origin has been proposed. It was suggested that *PCA3* originated from an ancient virus sequence that was incorporated into the human genome and therefore could be regulated by virus-specific patterns [8]. According to this report, the presence of additional features in the *PCA3* gene could corroborate this hypothesis. First, *PCA3* initial gene portion is included in a long interspersed nuclear element type 2 (LINE-2) repeat, a retrotransposon element derived from an ancient virus, which is also the case of the lncRNA *HULC*, regulated by hepatitis B virus and whose initial portion is also embedded in a long-terminal repeat retrotransposon-like sequence [9]. In addition, it was proposed that the *PCA3* promoter does not contain any canonical transcription factor-binding site and lacks any sequence similarity with human promoters. Despite that, a recent report showed that SNAIL transcription factor binds to *PCA3* promoter through an E-box element, activating *PCA3* expression [10]. Furthermore, *PCA3* localization in the opposite strand of *PRUNE2* (Figure 1C) is also similar to the case of Epstein–Barr virus bidirectional transcription, with the opposite direction mainly associated with the transcription of noncoding and regulatory genes [11]. Moreover, *PCA3* adenosine deaminases acting on RNA (ADAR) mediated editing is also a post-transcriptional mechanism largely employed in the cellular responses to viruses [11]. Despite these intriguing speculative evidence, experimental data are still needed to validate this hypothesis.

## *PCA3* FUNCTIONAL ROLES IN PROSTATE BIOLOGY

**Figure 2:**
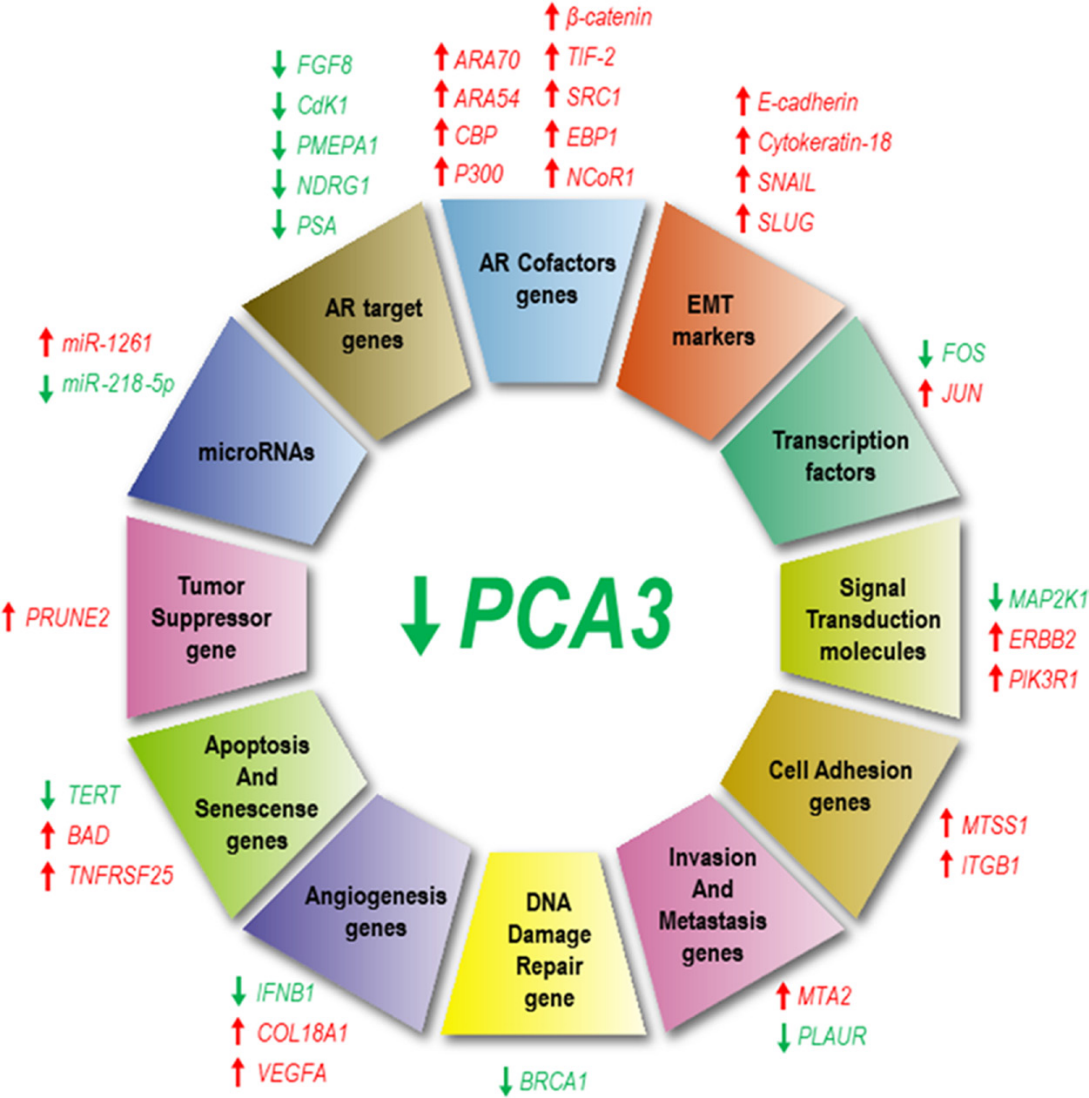
*PCA3* silencing modulates the expression of several genes and microRNAs. Genes that have been downregulated in response to *PCA3* silencing are represented by green arrows, while those upregulated are indicated by red arrows. Overview of different genes which expressions are modulated by *PCA3*, classified according to their functional roles: androgen receptor (AR) targets and co-factors, epithelial-mesenchymal transition (EMT), transcription, signaling transduction, cell adhesion, invasion and metastasis, DNA damage repair, angiogenesis, apoptosis and senescence, tumor suppression and miRNAs.

The description of *PCA3* roles in PCa tumor biology was pioneered and reported by our group [12]. Our investigation reported that transient knockdown of *PCA3* transcripts reduced cell growth and viability, in addition to inducing apoptotic cell death. These data reinforced the hypothesis that *PCA3* could modulate PCa cell survival. We also reported an association between *PCA3* and the androgen-receptor (AR) signaling pathway (Figure 2). We found that cells treated with AR agonist dihydrotestosterone (DHT) induced significant upregulation of *PCA3* expression, which was reversed by AR antagonist flutamide. In addition, we also observed upregulation of androgen-responsive genes (ARGs) (*TMPRSS2, NDRG1, GREB1, PSA, AR, FGF8, CdK1, CdK2*, and *PMEPA1*) in response to DHT treatment. Interestingly, these findings were reversed when silencing *PCA3* using RNA interference [12].

Some additional data have also indicated that *PCA3* and *PRUNE2* may have similar responsive mechanisms and evolutionary patterns [6]. However, regarding *PRUNE2* expression levels in response to androgen induction, these data are controversial, as follows. Clarke et al. showed that *PRUNE2* levels were induced by AR stimulation [6], whereas Salameh et al. observed that androgen stimulation decreased *PRUNE2* levels, besides inducing a concomitant increase in *PCA3* expression. Thus, *PRUNE2 PCA3* regulation appears to be sensitive to AR activation, one of PCa molecular hallmarks [13].

Salameh et al. showed a functional relationship between *PCA3* and *PRUNE2*, by demonstrating that *PCA3* modulated *PRUNE2* expression [13] (Figure 2). These authors proposed that this modulation could be mediated by ADARs. In this proposed mechanism, *PCA3* bound to *PRUNE2* pre-mRNA forms a double-stranded RNA with *PRUNE2*, which is then ligated to ADAR proteins, that in turn regulate *PCA3* and *PRUNE2* levels [13]. These enzymes are required for a co-regulatory effect on both RNAs, where *PCA3* negatively regulates *PRUNE2* levels and vice-versa (Figure 2).

It has been demonstrated that *PCA3* and *PRUNE2* display opposite roles in both *in vitro* and *in vivo* models of PCa [13]. These authors described that *PCA3* silencing or *PRUNE2* ectopic overexpression decreased cell proliferation and transformation *in vitro*. In addition, PCa cells overexpressing *PRUNE2* presented decreased cell adhesion, spreading and migration, while PCa cells in which *PRUNE2* was stably silenced presented larger tumor xenografts. These data showed the tumor suppressor activity of *PRUNE2*. In conclusion, silencing *ADAR1* in LNCaP PCa cell line, which increased *PRUNE2* expression as opposed to decreased *PCA3* levels, reduced tumor cell proliferation *in vitro* and *in vivo*, further demonstrating a functional role for the *PRUNE2*/*PCA3* regulatory axis in PCa [13].

A later report by our group [14] presented further comprehensive mechanisms for LNCaP cell line survival rates modulated by *PCA3*. We found that LNCaP cells in which *PCA3* was knocked-down induced the expression from 16 out of 84 tested tumor markers, including those involved in transcription control, cell signaling, angiogenesis, apoptosis, cell senescence, invasion, metastasis, cell adhesion and DNA damage repair [14] (Figure 2). These data indicated that *PCA3* modulated PCa cell survival through regulating the expression of key cancer-related genes (Figure 2), mainly those involved in controlling gene expression and cell signaling. *PCA3* knockdown also induced a significant upregulation of the cofactors *ARA70, ARA54, CBP, P300, β-catenin, TIF-2, SRC1, EBP1, and NCoR1*. These data further proposed that the upregulation of AR cofactor transcripts could be one of the possible mechanisms by which ARGs are negatively modulated in response to *PCA3* silencing [14]. Furthermore, *PCA3* modulated the expression of some EMT markers (*E-cadherin, cytokeratin-18, SNAIL* and *TWIST*), with some evidence that *PCA3* downregulation does not induce a complete reversion in the expression of epithelial and mesenchymal markers, which was compatible with a partial EMT (Figure 2). Moreover, by stable silencing *PCA3* transcript, PCa cell viability was lost [14], as observed when using *PCA3* transient silencing [12]. These data supported the proposal of *PCA3* knockdown as a putative therapeutic approach to inhibit PCa growth [14]. Further evidencing the key role of *PCA3* on PCa cell survival, it was reported that *PCA3* silencing sensitized PCa cells to enzalutamide-induced loss of cell growth, reinforcing the link between *PCA3* and modulation of AR signaling [15].

### *PCA3* and microRNAs

The interaction between lncRNAs, including *PCA3*, and microRNAs (miRNAs) can influence post-transcriptional regulation, as lncRNAs can act as competing endogenous RNAs [16]. LncRNAs have miRNA responsive elements, to which miRNA binds and sponges, controlling miRNAs endogenous availability to bind to their own mRNAs targets, thus affecting their expression [17]. In a recent report, a search for miRNAs presenting base pairing with *PCA3* was performed and 14 miRNAs were identified. Among further validated miRNAs, *miR-1261* was differentially expressed in response to *PCA3* knockdown. In addition, the overexpression of *miR-1261* induced *PCA3* downregulation, evidencing a regulatory effect between *PCA3* and *miR-1261* [10] (Figure 2).

It was also suggested that *PCA3* could target *miR-218-5p* [18], a miRNA that negatively regulates PCa cell invasion, proliferation [19], migration [20] and tumor angiogenesis [21]. *PCA3* knockdown decreased *miR-218-5p* expression levels *in vitro* and *in vivo* (Figure 2) and *miR-218-5p* suppressive effects over PCa biological functions are related to high mobility group box 1 (HMGB1) protein repression, suggesting that the *PCA3*-*miR-218-5p*-HMGB1 axis could be important for PCa progression [18]. Of note, the *miR-218-5p* is also targeted and regulated by other lncRNAs, such as *MALAT1* and *CCAT1* [22, 23].

## *PCA3* AS A BIOMARKER

Since its first description, *PCA3* has been especially investigated due to its major overexpression in PCa cells. This paramount attribute has highlighted the importance of further evaluation of its potential clinical usefulness. Therefore, *PCA3* molecular tests have been proposed, based on its detection by quantitative real-time PCR (qPCR), aiming to detect PCa cells in body fluids and urinary sediments, after digital rectal examination (DRE) [24, 25]. Since 2012, *PCA3* was approved as an auxiliary biomarker in the molecular diagnosis of PCa in the European Union, Canada and the United States [26]. Table 1 summarizes the reports on this issue.

**Table 1:** Main findings of *PCA3* as a marker for diagnosis, prognosis and active surveillance (AS)

Study	Assessment	Main findings
	**Diagnosis**	
[28]	Urine specimens (DNA, RNA, protein, and metabolites); *PCA3*	Non-invasive urine-based testing represented a rich source of novel biomarkers for PCa
[29]	Blood and urine specimens (mRNA); *PCA3* and *TMPRSS2:ERG*	Serum and urine molecular biomarkers have been identified including *PCA3*, which was introduced clinically
[30]	Urine specimens (mRNA); *PCA3*	The APTIMA *PCA3* assay added specificity to the algorithm for PCa
[31]	Biopsy specimens (mRNA); *PCA3*	*PCA3* as a first-line screening test showed improved performance
[33]	Urine specimens (mRNA); *PCA3*	*PCA3* score was highly correlated with the risk of having cancer on re-biopsy, and could prevent unnecessary prostate biopsies
[35]	Urine and tissue specimens (mRNA); *PCA3* and *PSA*	The quantitative RT-PCR assay for *DD3^PCA3^*, bringing great promise for molecular urine analysis, reducing the number of unnecessary biopsies
[47]	Urine specimens (mRNA); *PCA3*	Chronic prostatitis did not influence the *PCA3* score
[51]	Urine specimens (mRNA); *PCA3*	*PCA3* density showed a significant increase in specificity when compared with *PSA*, PSAD and *PCA3*
[54]	CTC in blood (mRNA) *PCA3*	A chip-based device platform using *PCA3* mRNA as a target to capture CTC was developed
[61]	Urine specimens (mRNA); *PCA3* and *TMPRSS2:ERG*	Urinary testing for *TMPRSS2:ERG* and *PCA3* could avert unnecessary biopsy
[62]	Peripheral blood (mRNA); *PCA3*, *PSA* and *hK2*	Combining *PCA3*, *PSA*, and *hK2* showed better performance than individual biomarkers alone in predicting PCa
[63]	Urine specimens (mRNA) *PCA3* and *PSA*	The combination of *PCA3* with *PSA* gives better overall performance in identification of PCa than serum *PSA* alone in the high-risk population
[64]	Urine specimens (mRNA); *PCA3*, *PSMA* and *PSGR*	*PSMA*, *PSGR*, and *PCA3* scores were significant predictors of PCa using a multiplex model
	**Prognosis and Active Surveillance (AS)**	
[68]	Blood and urine specimens (mRNA) *PCA3*, PHI and sarcosine	*PCA3*, PHI and sarcosine have been identified as predictors of PCa characteristics at final pathology
[74]	Urine specimens (mRNA); *PCA3*	*PCA3* provided incremental prognostic information in the AS setting
[75]	Urine specimens (mRNA); *PCA3*	The urinary *PCA3* test predicted Gleason grade re-classification amongst patients receiving a 5ARI during AS for low-risk PCa
[76]	Urine specimens (mRNA); *PCA3*	The prognostic significance of *PCA3* was confirmed as associated with tumor volume and Gleason score
[81]	Urine specimens (mRNA); *PCA3* and *TMPRSS2-ERG*	Urinary *PCA3* and *TMPRSS2-ERG* scores did not appear to be useful in assessing response to ADT in advanced PCa
[82]	Urine specimens (mRNA); *PCA3*	Dutasteride effect on the *PCA3* score was variable
[83]	Peripheral blood specimens (DNA); *PCA3*	The presence of the (TAAA)n STR polymorphism in the *PCA3* promoter region may be a risk factor for PCa in the Chinese population
[85]	Peripheral blood mononuclear cells (DNA); *PCA3*	The occurrence of a STR polymorphism might be related to the mutations of *PCA3* upstream loci
[86]	FFPE tissue blocks (DNA); *PCA3*	A TG dinucleotide repeat in *PCA3* was significantly associated with PCa risk and aggressiveness
[89]	Blood samples (DNA); *PCA3*	Carriers of the polymorphism *PCA3* -845 G > A had a higher risk for metastatic PCa

Abbreviations: 5-ARI: 5-Alpha Reductase Inhibitors; ADT: androgen deprivation therapy; AS: active surveillance; CTC: circulating tumor cells; FFPE: formalin-fixed paraffin embedded; hK2: human kallikrein 2; mRNA: messenger RNA; PCa: prostate cancer; PCA3: prostate cancer antigen 3; PHI: prostate health index; PSA: prostate specific antigen; PSAD: prostate specific antigen density; PSGR: prostate specific G protein coupled receptor; PSMA: prostate-specific membrane antigen; RT-PCR: reverse transcription polymerase chain reaction; STR: short tandem repeat.

### *PCA3* as a diagnostic biomarker

Considering current prostate-specific antigen (PSA) limitations as a biomarker for PCa [27], new PCa biomarkers have been proposed to improve the accuracy of PSA in the management of early PCa, including *PCA3*. Different from PSA, *PCA3* expression levels seem to be independent of patient age, inflammation, trauma or prior biopsies [28] and can be detected and quantified in urine [29]. The urinary *PCA3* as a test to detect PCa was approved by the US Food and Drug Administration (FDA) and is available to be used in private hospitals and clinics for PCa diagnosis [30]. It is a urine test, followed by a rectal examination, to facilitate the PCA3 to go into the urine. However, its utility as a first line test or to detect high-grade PCa disease remains controversial since some authors found a low sensitivity of using *PCA3* as biopsy indicator. It has been discussed that the *PCA3* score would fail to detect 36% of advanced cases in men with low PSA values [31].

The combination of DRE and PSA allows a correct PCa risk stratification in the majority of cases, although a significant proportion of indeterminate results lead uncertain physicians to perform a prostate biopsy [32]. Thus, *PCA3* test has been useful in clinical trials to guide the decision of those patients that will perform biopsy or re-biopsy after an initial negative biopsy with continued suspicion of PCa [33]. In this context, the Progensa *PCA3* test is a commercial available kit approved by the US FDA for men ≥ 50 years old with a previous negative biopsy and a persistent elevated PSA level to aid in decision-making regarding repeated biopsies [34].

*PCA3* transcript levels are measured through qPCR by Progensa test in urine samples obtained after a prostate massage in order to achieve the maximum number of prostatic cells. This measurement must be performed together with the *PSA* transcript, which has similar expression levels in tumor and benign cells. Thus, a *PCA3* score based on the ratio of *PCA3* to PSA transcripts can be determined [35].

The thresholds used for the *PCA3* score are still controversial. Several groups used a *PCA3* score threshold ≥ 35 [34, 36-41], while others explored values ≤ 35 [37, 40, 42–45] (Table 2). For instance, recent studies have demonstrated that a PCA3 score of 35 provides an optimal balance between sensitivity and specificity in diagnosing PCa with greater diagnostic accuracy than free/total PSA (cut-off 25%) [36, 38]; on the other hand, a PCA3 score threshold lower than 25 could be predictive of pathological indolent PCa [42].

**Table 2:** Outcomes for different PCA3 thresholds

Study	Threshold	Threshold relevance
[37]	50	Identification of men at high risk of harboring significant PCa who are candidates for RP
[39]	47	Providing a correlation between PCA3 score and tumor volume
[34, 38]	35	Optimal balance between sensitivity of 58% and specificity of 72%
[36]	35	Increased risk of PCa
[41]	35	Optimal balance with sensitivity of 54% and specificity of 74%
[40]	24 and 35	Independent predictors in multivariable intermediate-and high-grade disease models
[42]	25	Predictive of pathological indolent PCa
[43]	25	Optimal balance between sensitivity and specificity, and also NPV is greater enhanced
[44]	25	Effective to determine which men are candidates for AS
[37]	20	Selecting men with clinically insignificant PCa in whom AS may be appropriate
[45]	17	Increment in PA to detect PCa

Abbreviations: RP: radical prostatectomy; PCa: prostate cancer; NPV: negative predictive value; AS: active surveillance: PA: predictive accuracy.

*PCA3* score has been quite discussed, especially regarding the application to clinical practice. It seems practical to establish thresholds for guiding repeated biopsy decisions. Both the USA and European repeated biopsy studies have indicated that a *PCA3* score threshold of 35 could provide an optimal balance between sensitivity and specificity for detecting PCa [38]. Many studies have also reported that the *PCA3* score has been correlated to PCa significance. Some authors have found that the mean *PCA3* score was lower in men with indolent PCa than in those with clinically significant PCa, both in men with a positive biopsy and in those undergoing radical prostatectomy (RP) [37]. Other studies also found that *PCA3* score was higher in biopsies presenting GS ≥ 7, than in those samples with GS <7 [36, 44]. Furthermore, higher *PCA3* score was significantly correlated with larger tumor volume [44], while the median score was correlated with tumors presenting extracapsular extension [39]. Although these data demonstrate that *PCA3* score is related to PCa significance, there are three studies that were not able to show this relationship [46–48]. The association between *PCA3* levels and PCa significance should be further investigated.

*PCA3* has been shown to be PCa specific, since its expression is not influenced by other clinical conditions, such as chronic prostatitis, on the contrary of PSA levels. In such a situation, urinary *PCA3* test has been majorly recommended as a valuable tool to better identify those patients who really need to perform prostate biopsies. *PCA3* score was found to be negative (less than the usual threshold of 35) in a series of 38 patients, suggesting that *PCA3* test can be used as a valuable tool in patients with raised PSA levels and suspicion of chronic prostatitis, in order to distinguish those patients who will really benefit from prostate biopsy [49]. Such data are in agreement with other reports, in which it was demonstrated that *PCA3* score was similar in patients with benign prostatic hyperplasia (BPH) and/or normal parenchyma at biopsy in a large series with chronic prostatitis and high-grade prostatic intraepithelial neoplasia (HG-PIN) [50].

Several efforts have been made in order to improve the detection of PCa prior to biopsy by using additional approaches based on *PCA3* evaluation, such as the application of *PCA3* density evaluation [*PCA3*D: the ratio of urinary *PCA3* score/prostate volume (PV)]. Many authors have clearly established a correlation between tumor volume, as assessed from RP specimens, and *PCA3* score [40, 44, 51]. Some authors postulated that reporting *PCA3* score in relation to total PV could be of diagnostic interest, since the ratio *PCA3*D, would represent the proportion of PV occupied by the tumor [52]. In this study, they found higher diagnostic accuracy for *PCA3* score and *PCA3*D than PSA and PSAD (ratio of urinary PSA score/PV). Corroborating these data, others also showed a significant increase in PCa diagnostic specificity using *PCA3*D when compared to PSA, PSAD and *PCA3* score. These authors concluded that *PCA3*D could be used as a mini-nomogram with a 70% risk of positive initial biopsy when *PCA3* score > PV [53].

A prospective study including 594 samples addressed for initial prostate biopsy and *PCA3*-based nomograms tests provided significant predictive accuracy for *PCA3* score. The urinary *PCA3* test and the *PCA3*-incorporating nomograms can be considered as additional reliable tools to support the initial biopsy decision [54].

However, certain limitations regarding the use of *PCA3* molecule should be considered. As a typical RNA molecule, which have sequences that contain the active RNA degradation systems and therefore present defects in processing, folding, or assembly with proteins, being rapidly degraded by the surveillance machinery, PCA3 is unstable and its capture and preservation needs to be accurate. Aiming to circumvent this limitation, some approaches have been tested in order to improve higher yields of RNA extraction, such as commercially available magnetic beads (Mag-Cap), phenol-chloroform, affinity columns or Mag-Cap for RNA extraction in urine samples [55].

Another issue of widespread interest for clinicians is the capture of circulating tumor cells (CTC) from the blood. In this context, Sioss et al. [56] developed a platform using RNA purified by enriched CTCs from blood samples, using *PCA3* as target. The platform consists of a chip-based device, which utilizes antisense oligonucleotides attached to silica-coated nanowires (NWs) to detect *PCA3*.

More recent, combinations between *PCA3* and other biomarkers are emerging, including *TMPRSS2:ERG* fusion [57–61]. *TMPRSS2* (androgen-regulated transmembrane protease serine 2), is fused to the ETS-related gene (*ERG*). A truncated ERG protein is overexpressed following androgenic stimulation of the *TMPRSS2* promoter [62]. The combination of these two biomarkers in the urine after DRE improved the specificity for detecting PCa with GS ≥ 7. The authors stressed that 42% of unnecessary prostate biopsies would have been avoided by using the urine assay results in order to select men to perform biopsy [63].

In an attempt to improve PCa diagnosis, some studies have demonstrated better results of *PCA3* in association with PSA [64, 65], in addition to prostate-specific G-protein coupled receptor (PSGR) [66], a biomarker previously described to be overexpressed in PCa tissue [67]. Other authors found that the combination of *PCA3*, PSA, and human kallikrein 2 (hK2) largely improved area under the curve (AUC)-receiver operating characteristic (ROC), especially those patients presenting PSA 4-10 ng/mL [64]. PCa was quantitatively detected through overexpressed *PCA3* and PSA genes, in urine sediments of men with PCa or BPH, after prostatic massage. The aforementioned markers combined had a sensitivity of 80.2% and a specificity of 100 % [68].

Recent data also revealed that a logistic regression algorithm combining *PCA3* with PSA significantly increased PCa diagnostic properties [65]. This combined evaluation was able to discriminate low-grade from high-grade cancers. These data suggest that *PCA3* improves the diagnostic sensitivity and specificity of PSA and that the combination of *PCA3* with PSA analysis provides better overall performance in identification of PCa than serum PSA evaluation alone in the high-risk population.

A multiplexed qPCR assay, using the combination of the three overexpressed genes in PCa, which are prostate-specific membrane antigen (PSMA), PSGR and *PCA3* on urine sediments from patients who were indicated for prostate biopsy, provided an improve on the predictive ability when compared to the same test using *PCA3* or PSA genes alone [66]. These results further indicated the clinical usefulness of the *PCA3* and PSA combination as better approaches in the early diagnosis of PCa.

### *PCA3* in prognosis and active surveillance (AS)

*PCA3* evaluation has also been proposed for monitoring and predicting PCa clinical outcome, which would likewise aid in treatment decision strategies. An increasing body of evidence will be discussed herein showing that *PCA3* levels have been associated with PCa aggressiveness. Despite that, there are also contradictory reports that fail to report the correlation of *PCA3* levels and PCa outcomes. Additionally, one of the proposed clinical utilities of *PCA3* is its use in approaches that could screen those patients that could benefit from active surveillance (AS), a conservative management option for men with low-risk PCa that ought to decrease overtreatment. The majority of AS protocols require serial prostate biopsies, associated with patient discomfort and risk of complications [69].

It was reported that *PCA3* levels, as well as Prostate Health Index (PHI) and sarcosine levels, were positively associated with some prognostic markers, as tumor volume ≥0.5 mL, pathologic GS ≥7 and pT3 disease [70]. In addition, other authors showed that adding PHI and *PCA3* to the AS inclusion criteria of the contemporary Epstein and the Prostate Cancer Research International: Active Surveillance (PRIAS) protocols improved their prognostic performance to predict the presence of pathologically insignificant prostate cancer, in a retrospective study with patients who underwent RP but would be eligible for AS [71].

The prognostic validity of *PCA3* and TMPRSS2:ERG fusion transcripts, in combination, have been investigated, which showed their potential to reduce unnecessary prostate biopsies and guide risk stratification, besides being more specific than serum PSA [72]. It was also reported that *PCA3*, *TMPRSS2:ERG* and PHI were predictors of a tumor volume ≥0.5 mL, although multifocality was only predicted by *PCA3* score, in patients who underwent RP for biopsy-proven PCa [73].

Moreover, high *PCA3* scores in urine have been significantly correlated with a high GS, percentage of positive biopsy cores and advanced clinical stage [74]. An additional report also showed that the *PCA3* score values were associated with greater tumor aggressiveness, as measured by correlation with GS [75]. In another investigated AS cohort, it was shown that men with GS reclassification (GR) had higher first *PCA3* (f*PCA3*) and subsequent *PCA3* (s*PCA3*) levels [76]. Additionally, a prospective phase II study described that the *PCA3* test predicted the risk of GR in patients at low-risk PCa patients, in AS, complemented with 5-alpha-reductase inhibitor prescription [77]. Further, a recent report confirmed the prognostic value of the *PCA3* score, which was positively associated with PCa tumor volume and GS [78].

Conversely, it is important to mention that some analysis were unsuccessful in showing *PCA3* validity for prognostic use. Some authors demonstrated that *PCA3* presented low sensitivity and high false negative rates for predicting high GS in initial biopsy (GS ≥8). Additionally, in patients submitted to RP, low *PCA3* levels were associated with adverse pathological features in RP, clinical recurrence outcome and the greater probability of metastatic progression [79]. Moreover, in a cohort of patients in AS, *PCA3* was not an independent predictor of PCa diagnosis on repeat biopsies [80]. Another report also found no correlation between *PCA3* score and GS on biopsy or clinical tumor stage, although it was presented as a valuable diagnostic biomarker for PCa [81]. Other authors showed no correlation between *PCA3* score and GS or pathological stage of prostatectomy samples, even though it was associated with total tumor volume, apical and basal invasion, bilaterality and multifocality [82].

Hence, the potential use of *PCA3* as a prognostic biomarker is still under investigation and discussion. Clinical evidence is yet at early stages to consider this transcript as a biomarker candidate for PCa prognosis and further efforts are needed to elucidate this question. Also, some investigations demonstrated that *PCA3* levels in the urine may be significantly affected by androgen deprivation therapy, which would restrict the consideration of *PCA3* levels to monitor PCa clinical course [83, 84].

## *PCA3* DETECTION IN EXOSOMES – POTENTIAL USE

**Figure 3:**
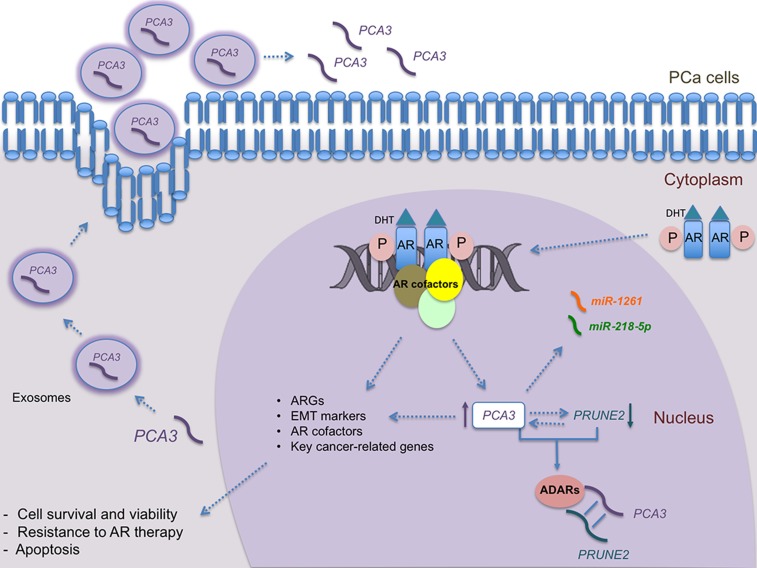
Overview of *PCA3* roles in androgen responsive PCa cells. *PCA3* transcript, which can be detected both into the nucleus and the cytoplasm, is regulated by androgen signaling. Androgen/DHT binds to the AR promoting its phosphorylation, which leads to its dimerization and translocation to the nucleus. Then, phosphorylated AR binds to the promoter region of target genes, activating their expression, including *PCA3*. *PCA3* also modulates the expression of several key cancer-related genes, including ARGs, AR cofactors, EMT markers, and *PRUNE2*. The binding of *PCA3* to *PRUNE2* pre-mRNA forms a double-stranded complex, which is then linked to ADAR proteins, that in turn regulate *PCA3* and *PRUNE2* levels. Moreover, *PCA3* negatively modulates *PRUNE2* expression and vice-versa. Furthermore, *PCA3* can modulate the availability of some miRNAs, such as *miRNA-1261* and *miR-218-5p*, by base pairing with them. In addition to classical overexpression of *PCA3* in body fluids and urine, *PCA3* transcript has also been detected in exosomes, from which it can be delivered into the extracellular environment. DHT: dihydrotestoterone; *PRUNE2*: Prune Homolog 2 Coding; PCa: Prostate Cancer; *PCA3*: Prostate Cancer Antigen 3; miR: microRNAs; ADAR: Adenosine Deaminases that act on RNA; AR: Androgen Receptor; ARGs: Androgen-Responsive Genes; EMT: Epithelial-Mesenchymal Transition.

The exosomes are extracellular vesicles secreted from prostate non-tumoral and malignant cells present in a variety of body fluids (blood, urine, semen and prostatic fluid). Prostate and cancer-associated markers are also present in urinary exosomes [85]. Because multiple exosomes have been associated with both PCa and elevated GS, there is increasing interest in investigating the exosomes as a potential source of biomarkers for PCa [86]. In this scenario, *PCA3* has been detected in urinary exosomes, in which their content is protected from degradation [87]: (Figure 3). The levels of *PCA3* and ERG prostate-biomarkers have been compared in urine samples and it was found that the whole urine could be the substrate for PCa diagnosis. It has also been found that among various cancer-related genes, *PCA3* could differentiate biopsy positive patients from those negative using RNA isolated from exosomes [88]. Although the results are promising, larger studies are needed to confirm the potential clinical usefulness of *PCA3* detection in exosomes, which must be prospectively assessed in larger clinical cohorts.

## *PCA3* EXPRESSION IN NON-PROSTATIC TISSUES

*PCA3* transcript expression has been mainly associated with prostate tissues, and it had been proposed for quite some time that it was a prostate-specific gene product [1, 89]. However, the expression of this lncRNA has been recently described in other tissues and pathological conditions. The detection of *PCA3* in ovarian healthy and cancer tissues, as well as in ovarian cancer cell lines, has been described [90]. *PCA3* knockdown in ovarian cancer cells led to the suppression of cell migration, invasion and viability, besides induction of G1 cell cycle arrest and apoptotic cell death. The same report also proposed that the *PCA3* gene 3’UTR region presented a potential binding site to miR-106b and that some genes regulated by this miRNA are also affected when *PCA3* expression is suppressed. Nevertheless, further data are needed to understand the mechanisms modulating *PCA3* expression in ovarian cancer cells [91].

*PCA3* expression has also been described in a recent study aiming to characterize differentially expressed RNAs in Parkinson and Alzheimer diseases. *PCA3* was found to be upregulated in cerebrospinal fluid exosomes extracted from both diseases, as compared to healthy controls [92]. These findings suggested that *PCA3* may also participate in the pathogenesis of such other diseases, and its use as a potential biomarker in these conditions should be further explored.

## FUTURE PERSPECTIVES AND DIRECTIONS

In the last decade, an exciting boom of experimental research has brought to light the pivotal biological functions of lncRNAs, representing more than half of the non-coding transcriptome, along with their dysregulation in many diseases, including cancer [93]. In this context, *PCA3* has been extensively studied since 1999, showing its involvement on modulating PCa survival, link to AR signaling, besides its relation to *PRUNE2* expression and the potential ability to modulate the expression of key cancer-related genes. These known roles are summarized in Figure 3. However, there are still open questions to further understand its role in PCa biology, especially how its expression is controlled by AR signaling and its potential link to resistance to currently proposed therapies focused to these pathways. New studies should be devoted to investigate *PCA3* roles regarding potential utility in immunotherapy and its relation to immune system evasion during PCa development. Given the recent findings of *PCA3* in other tumor and pathologies, additional studies should better comprehend *PCA3* expression patterns and the factors that modulate *PCA3* aberrant expression in pathological conditions.

Altogether, these data showed that *PCA3* is able to modulate distinct signaling pathways, mostly those involved on cell survival [15]. Recent evidence also points *PCA3* as a regulator of transcriptional levels, mediated by ADARs [13], being able to target miRNAs [18] and possibly a precursor of several miRNAs, as has been shown for other lncRNAs [94]. The lncRNAs act as competing endogenous RNAs (ceRNAs), where RNA molecules regulate each other through their biding sites, triggering decay of the targeted lncRNAs [95]. We then speculate *PCA3* as a key regulator of gene expression by binding to several types of RNA molecules, especially those aberrantly expressed in pathological conditions. Once PCA3 has also been detected in other diseases beyond cancer, such as Alzheimer and Parkinson [92], and there is rising recognition that lncRNAs have been implicated in these processes [95], we then postulate that *PCA3*, when aberrantly expressed in these diseases could post transcriptionally regulate the main regulatory pathways mediating these processes. All these possibilities open new avenues to target *PCA3* therapeutically to combat these pathological conditions.
